# Aspects histo-épidémiologiques des cancers génitaux de la femme dans la région du Littoral, Cameroun

**DOI:** 10.11604/pamj.2015.21.116.6755

**Published:** 2015-06-12

**Authors:** Jean Paul Ndamba Engbang, Valère Mve Koh, Charlotte Nguefack Tchente, Amadou Fewou

**Affiliations:** 1 Faculté de Médecine et des Sciences Pharmaceutiques de l'Université de Douala, Cameroun; 2 Hôpital Laquintinie de Douala, Cameroun; 3 Centre Hospitalier Universitaire de Yaoundé, Cameroun; 4 Hôpital Général de Douala, Cameroun; 5 Faculté de Médecine et des Sciences Biomédicales de l'Université de Yaoundé I, Yaoundé, Cameroun

**Keywords:** Cancer, génital, féminin, épidémiologie, histologie, Cameroun, Cancer, génital, féminin, épidémiologie, histologie, Cameroun

## Abstract

Décrire les caractéristiques épidémiologiques et histopathologiques des tumeurs malignes génitales de la femme dans la région du littoral du Cameroun. Il s'agissait d'une étude rétrospective descriptive et analytique portant sur les cancers des organes génitaux de la femme, histologiquement prouvés pendant une période de 10 ans (2004-2013), répertoriés dans les registres des trois laboratoires d'anatomopathologie de la région (Hôpital Laquintinie de Douala, Hôpital Général de Douala, laboratoire Anapathos) et des services d'oncologie de ces hôpitaux. Les variables étudiées étaient: la fréquence, l’âge, le sexe, la localisation de la tumeur et le type histopathologique. Au total, 802 cas de cancers génitaux de la femme ont été recensés, soit une fréquence annuelle de 80,2 cas en moyenne. Le col utérin avec 580 cas (72,32%) a été la localisation la plus fréquente; suivi de l'endomètre (corps utérin) avec 93 cas (11,60%), puis des ovaires 91 cas (11,35%). L’âge moyen des patientes était de 50, 30±12,67 ans, avec les extrêmes allant de 14 à 85 ans. Selon le type histologique, les tumeurs épithéliales ont été les plus fréquemment rencontrées, soit 758 patientes (94,51%), les lymphomes venaient en seconde position avec 29 cas (3, 62%), les autres variétés histologiques (sarcomes, tumeurs germinales, tumeurs du mésenchyme et du cordon) représentant moins chacune de 1%. Les tumeurs malignes des organes génitaux féminins sont fréquentes dans la région du littoral du Cameroun, elles sont dominées essentiellement par le cancer du col utérin. Les tumeurs épithéliales sont le type histologique le plus fréquent.

## Introduction

Le cancer a été longtemps, à tort, considéré comme une pathologie occidentale, à cause de l'ignorance des données statistiques. ces dernières ont montré que, ce fléau serait non seulement la 3^è^ cause de mortalité dans nos pays, mais que 72% des décès mondiaux dus au cancer sont enregistrés dans les pays en voie de développement [1, 2]. En Afrique sub-saharienne, le cancer touche particulièrement la population jeune. Les femmes sont les plus exposées; elles représentent jusqu’à 68% des cas [3]. Selon les dernières données au Cameroun sur les cancers gynécologiques, le cancer du col utérin occupait la deuxième place après celui du sein [3]. Mais, ces séries ne concernaient que la ville de Yaoundé, capitale politique publiées du Cameroun. Nous n'avons retrouvé aucune publication concernant des données liées aux cancers féminins dans la région du littoral, premier pôle économique du Cameroun et dans laquelle se trouve Douala, la plus grande ville du pays. C'est pour cela que nous nous sommes proposés de faire ce travail qui pourra contribuer à une meilleure prise en charge de cette grave pathologie.

## Méthodes

Il s'agissait d'une étude rétrospective descriptive analytique portant sur les cas de cancers des organes génitaux féminins, diagnostiqués sur une période de 10 ans, de 2004 à 2013, dans les services et laboratoires d'anatomo-pathologie de l'Hôpital Général de Douala, Hôpital Laquintinie de Douala et du laboratoire Anapathos, des centres de référence de diagnostic des cancers dans la région du littoral. Les cas ont été colligés à partir des registres et des dossiers des patients de ces centres. Le matériel d’étude était constitué de biopsies et des pièces opératoires fixées au formol 10% et traités selon les règles conventionnelles en anatomopathologie. Les variables étudiées étaient l’âge, le sexe, la localisation de la tumeur, le type et le grade histologique, était-ce pour mettre en évidence un gène, à ce moment-là l'expression du gène devient la variable. L'analyse des variables a été réalisée avec le logiciel Statistical Package for Social Sciences (SPSS), version 16.0.

## Résultats

### Généralités

**Figure 1:**
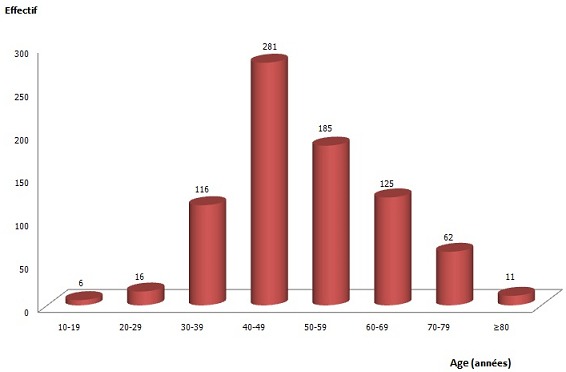
Répartition des cas de cancers selon l’âge

Durant la période d’étude, nous avons retrouvé 802 cas de cancers des organes génitaux féminins, soit une fréquence annuelle de 80,2 cas. 580 cas (72,32%) de cancers du col utérin, 93 cas (11,60%) de cancers de l'endomètre, 91 cas (11,35%), ont concerné les cancers de l'ovaire. Les tumeurs épithéliales ont été les types les plus fréquents avec 758 patientes (94,51%), suivis des lymphomes avec 29 cas (3, 62%) (Tableau 1). L’âge moyen des patientes etait de 50,30±12,67ans, avec les extrêmes de 14 et 85 ans. L'incidence la plus élevée a été observée dans l'intervalle de 40-49 ans. Au sein de la population féminine en âge de procréer, l'on a retrouvé 419 cas (52, 24%), contre 383 cas (47,76%) pour les femmes de 50 ans et plus. On a note une augmentation progressive avec un pic dans l'intervalle 40-49 ans (Figure 1).

**Tableau 1:** Répartition des groupes histologiques en fonction des sites

Sites	Types histologiques						
	Tumeurs	Tumeurs	Tumeurs	T secondaires			Total
	épithéliales	germinales	MC	LMNH	Sarcome	Autres T	
Col	568	-	-	8	4	-	580
Ovaires	62	3	2	20	1	3	91
Corps	92	-	-	1	-	-	93
Vagin	13	-	-	-	2	-	15
Vulve	11	-	-	-	-	-	11
Trompes	12	-	-	-	-	-	12
Total	758	3	2	29	7	3	802
%	94,51	0,37	0,25	3,62	0,87	0,37	100,00

**CE : Carcinome épidermoide, ADK :Adénocarcinome, CI :Carcinome Indifférencié, CAS : Carcinome Adénosquameux, LMNH :Lymphome Malin Non-Hodgkinien**

### Le col de l'utérus

Durant la période d’étude, 580 cas (72,32%), de cancers du col de l'utérus ont été diagnostiqués, premier organe atteint. L’âge moyen était de 51,22±11,93 ans, les extrêmes allant de 17 à 82 ans. Les tumeurs épithéliales ont été rencontrées chez 548 patientes (94,48%). Au total, 418 cas de carcinomes épidermoides ont été recensés et 130 cas d'adénocarcinome (Tableau 2).

**Tableau 2:** Répartition des types histologiques du cancer utérin en fonction de l’âge

Ages	Types histologiques						
	CE	ADK	CI	CAS	LMNH	Sarcome	Total
20-21	2	1	1				4
30-39	60	16	1	1			78
40-49	148	60	3	4	1	3	219
50-59	89	36	3	2	2	3	135
60-69	73	10	2	1	1		87
70-79	40	5	2			1	48
≥80	6	2				1	9
Total	418	130	12	8	4	8	580
**%**	72,07	22,41	2,07	1,38	0,69	1,38	100

### Le corps utérin (endomètre)

**Figure 2:**
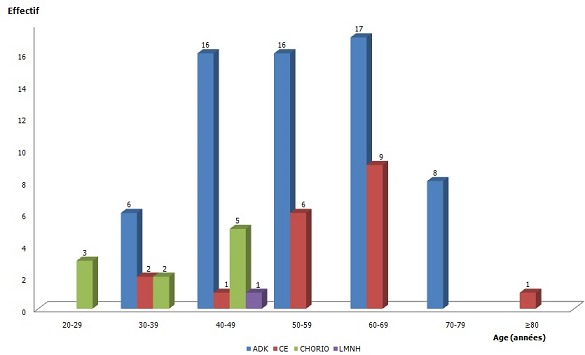
Répartition des types histologiques du cancer de l'endomètre suivant l’âge

Le cancer de l'endomètre a été retrouvé chez 93 patientes (11,60%). C'est le deuxième organe le plus atteint. La moyenne d’âge était de 53,72±13,32 ans, les extrêmes étant de 26 et 85 ans. Les types histologiques retrouvés étaient surtout les adénocarcinomes (ADK), 63 cas (67,74%) et les carcinomes épidermoides (CE) avec 19 cas (20,43%). La tranche d’âge la plus représentée était les 40-69 ans. 10 cas (10,75%) etaient des choriocarcinomes (Figure 2).

### Les ovairies

Au total, 91 cas (11,35%) de cancers des ovaires ont été retrouvés, faisant de cet organe le troisième le plus atteint. La moyenne d’âge des patientes était de 42,40±13,28 ans (extrêmes: 14-70 ans). Les tumeurs épithéliales sont concerné 62 cas (68,14%, suivies des autres tumeurs avec 24 cas (26,37%). Les adénocarcinomes (ADK Séreux) avaient à eux seuls 48,39% des cas des tumeurs épithéliales. Les lymphomes malins non-Hodgkiniens (LMNH) enregistraient 20 cas, parmi lesquels, 4 cas de Burkitt. Au total, 9 cas de tumeurs bilatérales avaient été identifiées (Tableau 3).

**Tableau 3:** Types histologiques des cancers ovariens en fonction de l’âge

AGE		10-19	20-29	30-39	40-49	50-59	60-69	70-79	TOTAL	%
**A**	ADK S	_	1	8	12	6	1	2	30	32,97
	ADK M	1	_	3	4	3	_	_	11	12,09
	ADK CC	_	_	2	3	1	_	_	6	6 ,59
	C I	_	_	_	2	_	1	_	3	3,30
	ADK NP	_	1	2	3	5	_	1	12	13,19
**B**	C E	2	_	_	_	_	_	_	2	2,20
	Tératome Malin	_	1	_	_	_	_	_	1	1,10
**C**	T G	_	_	_	_	_	1	_	1	1,10
	Androblastome	_	_	_	_	_	1	_	1	1,10
**D**	LMNH	3	4	4	4	4	1	_	20	21 ,98
	Sarcome	_	_	_	_	_	1	_	1	1,10
	ADK Endométrial*	_	_	1	1	1	_	_	3	3,30

**A: Tumeurs épithéliales-stromales de surface, B:Tumeurs germinales, C:Tumeurs du mésenchyme et des cordons sexuels, D:Autres tumeurs /secondaires. S:Séreux, M:Mucineux, CC:à cellules claires, CI:Carcinome indifféréncié, NP:non précisé, CE:carcinome épidermoide, TG:Tumeurs de la granulosa.**

* **Secondaire à un ADK endometrial**

### Les trompes

Sans la série, 11 cas de cancers tubaires ont été collectés. L’âge moyen de survenue s’établi établi à 48,36±9,94 ans, les extrêmes étant de 33 et 66 ans. L'adénocarcinome a été le seul type histologique retrouvé.

### Le vagin

L'on a recensé 15 cas de tumeurs malignes de cet organe, se qui représente 1, 87% des cancers de l'appareil reproducteur féminin. La moyenne d’âge de ces femmes était de 42,67±10,71 ans avec des extrêmes de 25 et 65 ans. Le carcinome épidermoide a été le type histologique le plus fréquent (60,00%- des casA) des cas, suivi de l'adénocarcinome, notamment celui à cellules claires qui a récolté 26,67% des cas et enfin les sarcomes avec 2 cas, un léiomyosarcome et un sarcome de Kaposi.

### Cancers de la vulve

Ces cancers avaient une fréquence de 1, 50% (n = 12) parmi les cancers génitaux de la femme. La moyenne d’âge était de 53,50±16,28 ans avec les extrêmes de 22 et 85 ans et un pic dans la classe d’âge de 50-59 ans. Les types histologiques rencontrés étaient: le carcinome épidermoide (75,00%) avec un cas de carcinome in situ, l'Epithelioma basocellulaire, la maladie de Bowen et l'adénocarcinome avec chacun 1 cas (8,33%).

## Discussion

L’âge moyen de nos patientes était de 50,30±12,67ans, avec les extrêmes de 14 et 85 ans. L'incidence la plus élevée a été observée dans l'intervalle de 40-49 ans. Nous avons constaté un aspect pyramidal de la fréquence avec un pic entre 40-49 ans, N'Dah et al. avaient noté la même répartition avec un pic entre 45-55 ans [4]. Nayama M et al. ont trouvé une nette augmentation de la fréquence à partir de 25 ans et la présence d'un pic important qui se situait entre 35-44 ans (32.5% des patientes) [5]. Cette période correspondant à l’âge de procréer, est le reflet de la période des activités génitales et de tous les événements qui en découlent (rapports sexuels précoces, infections sexuellement transmissibles, nombreuses grossesses) et surtout de l'insuffisance du programme de dépistage des cancers dans notre pays.

Le col utérin est la localisation la plus fréquente (72,32%). L’âge moyen était de 51,22±11,93 ans, de 17 à 82 ans. Cette proportion est inférieure à celle trouvée par N'Dah et al. qui évoquaient 82, 85% [4]. En zone urbaine au Cameroun (Yaoundé), le cancer du col représentait 13.8% de tous les cancers indépendamment du sexe, au second rang après le sein [3]. Ce qui rejoint la position d'autres auteurs ayant étudié les cancers gynécologiques et mammaires dans la capitale camerounaise et qui le classaient au second rang après le sein [6]. « Les pays en voie de développement comme le nôtre, sont très exposés à la survenue de ce cancer, car les facteurs de risque connus à savoir les rapports sexuels précoces (avant l’âge de 17 ans), les partenaires multiples, la profession itinérante du mari, les infections cervico-vaginales chroniques, les déficits immunitaires (HIV, transplantation rénale), les grossesses nombreuses et surtout les infections à HPV oncogènes (16,18, 31, 45), y sont très rencontrés [5, 7]. Mandelblatt et al. affirmaient que la combinaison des deux virus oncogènes (HIV et HPV) est responsable de l'amplification de la carcinogenèse avec le risque de développement précoce du cancer utérin [8]. La prédominance des tumeurs épithéliales et notamment des carcinomes épidermoides est confirmée par d'autres études [5]. Tous ces facteurs développés amènent à inciter à la vulgarisation des programmes de diagnostic précoce, de dépistage par l´inspection visuelle après application de l´acide acétique et la vaccination anti-HPV.

Dans notre série, le cancer de l'endomètre a occupé la deuxième place après celui du col utérin. Ce rang est retrouvé dans d'autres séries africaines [4, 5]. C'est le cancer gynécologique le plus fréquent dans les pays développés [9]. Avec une moyenne d’âge de 53,72±13,32 ans, notre étude a confirmé le constat fait par plusieurs auteurs selon lequel, le cancer de l'endomètre, tumeur hormonodépendante, est un cancer de la femme âgée en général: son incidence augmente significativement après 40 ans après 75 à 79 ans [9]. Le pic que nous avions observé entre 60-69 ans, reste faible par rapport aux données occidentales, où l'on l'observe entre 75 et 79 ans [10]. Certains auteurs attribuent cette différence à l'espérance de vie faible en Afrique et au fait que les données révélées ici sont sous évaluées car une bonne partie des femmes en post ménopauses vivent dans des zones rurales où les centres médicaux sont rares [4].

Le cancer des ovaires a été le troisième en fréquence dans notre étude. Certains auteurs trouvaient que ce cancer était le troisième de l'appareil reproducteur derrière ceux du sein et de l'utérus en Afrique sub-saharienne [2]. La moyenne d’âge des patientes ici était de 42,40±13,28 ans (extrêmes: 14-70 ans), chiffres proches des données de N'Dah et al. qui trouvaient un âge moyen de 40 ans avec un pic entre 45 et 54 ans [4]; toutefois, d'autres auteurs ont trouvé une moyenne d’âge proche ou supérieure à 50 ans [11, 12]. Parmi les facteurs de risque, on distingue les facteurs génétiques, les facteurs hormonaux et ceux liés à l'environnement au mode de vie [11, 13]. La théorie de Fathalla suggère que le risque de cancer de l'ovaire augmente avec le nombre d'ovulations, du fait que chaque cycle ovarien fait subir à l’épithélium de l'ovaire un traumatisme dont il devra cicatriser [14]. Les tumeurs épithéliales dominaient la structure histopathologique avec 62 cas (68,14%), valeur supérieure à celle qu'avait trouvée N'Dah et al., 57,48% [4]; toutefois, inférieure à celle d'autres auteurs [5, 11]. Nous avons noté 21,98% de lymphomes malins non-hodgkiniens, parmi lesquels celui de Burkitt. Le Cameroun fait partie de la ceinture africaine où ce type de lymphome sévit de manière endémique, ceci à cause de la prévalence élevée des infections à virus Epstein-Barr et du paludisme, pathologies reconnues comme étant des facteurs favorisant la survenue des lymphomes de Burkitt [15].

Le cancer des trompes a occupé la dernière place avec 11 cas. La moyenne d’âge était de 48,36±9,94 ans. Le cancer de la trompe utérine est rare, il représente moins de 1% de l'ensemble des cancers gynécologiques. Probablement sous estimé, car fréquemment diagnostiqué à tort comme une pathologie ovarienne, utérine, ou tubaire bénigne. Ces données peuvent avoir été faussées par les critères de définition qui attribuent à l'ovaire les formes prenant « en masse les deux organes » [16]. Ces difficultés diagnostiques expliquent d'une part le mauvais pronostic de ces tumeurs et d'autre part, la non codification du traitement. La moyenne d’âge trouvée est semblable à semble à celle de plusieurs autres auteurs, qui ont retrouvé un âge moyen autour de la cinquantaine [16, 17] et chez lesquels l'adénocarcinome restait le type histologique le plus retrouvé.

Le cancer du vagin a occupé le quatrième rang. Sa fréquence est estimée autour de 3% [9, 12], ce qui est supérieure à notre valeur. La moyenne d’âge de 42,67±10,71 ans retrouvée se rapproche plutôt des valeurs retrouvées par N'Dah et al. 42,93 ans [4]. Les facteurs tels que les conditions socio-économiques, le tabagisme les infections génitales, la multiplicité des partenaires sexuels joueraient un rôle non négligeable dans le développement de cette pathologie [12]. Le carcinome épidermoide a été retrouvé comme le type histologique le plus fréquent conformément à la littérature [4].

Dans notre étude, le cancer de la vulve a présenté une faible fréquence comme celle retrouvée dans la plupart des séries. La moyenne d’âge était de 53,50±16,28 ans avec les extrêmes de 22 et 85 ans. Dans la littérature, on s'accorde à définir cette pathologie comme celle du sujet âgé [18]. L'hypothèse virale a toujours été beaucoup plus évoquée concernant la survenue de ce cancer chez les jeunes, notamment l'HPV [19], alors que la carence oestrogénique expliquerait sa genèse chez la femme ménopausée [20].

## Conclusion

Les cancers génitaux de la femme sont fréquents dans la région du Littoral du Cameroun. Le cancer du col utérin est le plus rencontré. La possibilité de prévention nécessite qu'on y apporte une attention particulière, par la sensibilisation et la vaccination des jeunes filles. Les autres cancers gynécologiques sont présents.
